# Within-trial and across-trial distractor rejection mechanisms

**DOI:** 10.3758/s13414-026-03311-x

**Published:** 2026-07-20

**Authors:** Luca Betteto, Matteo De Tommaso, Massimo Turatto

**Affiliations:** 1 CIMeC, Center for Mind/Brain Sciences, University of Trento, Corso Bettini, 31, 38068 Rovereto, Italy; 2 Department of Psychology, University of Milano Bicocca, Milan, Italy

**Keywords:** Attentional capture, Distractor suppression, Distractor expectation, Surprising stimuli

## Abstract

Color singleton distractors’ interference is known to be modulated by rejection mechanisms based on the distractors’ probability of occurrence at different locations. Here, to further address color-singleton distractor rejection, we used a modified version of the additional-singleton paradigm, where four consecutive displays were presented in rapid succession on each trial. In Experiment 1 the irrelevant singleton was either presented in the last display (single location, 40% probability) or repeated in all four displays (repeated location, 10% probability), with the two conditions appearing at separate locations. Contrary to common observations, we found that capture was much more attenuated at the repeated location, despite the lower distractor probability. Furthermore, we found no signs of target-processing impairment at the repeated location where capture was basically eliminated, whereas capture was present at the single location. In Experiment 2, where both locations had repeated distractors, we found target-processing impairment only at the location with the highest distractor probability (40%). Experiment 3 indicated that suppression, regardless of any within-trial distractor repetitions, becomes evident only when the distractor probability is high enough (35%). Experiment 4 replicated the main findings in a single experiment, within the same group of participants. The results suggest the possible existence of distinct rejection mechanisms based on within-trial and across-trials distractor repetitions. While suppression is probably involved in the across-trials rejection mechanism, we propose that the one operating on a shorter within-trial time scale is likely controlled solely by a distractor-expectation or distractor-prediction error mechanism.

## Introduction

Attention is naturally attracted by salient stimuli, but when such stimuli are irrelevant for the organism’s current goal, they may become potential distractors, thus interfering with the task at hand.

Accordingly, it has been proposed that the presence of an irrelevant color singleton in the search display slows down response times (RTs) when participants are searching for a target singleton element. It has been debated whether attentional capture is completely stimulus driven (Theeuwes, 1992), or susceptible to some degree of top-down control (Bacon & Egeth, 1994; Folk et al., 1992), and while the debate between the different views is still alive (Luck et al., 2021), evidence has accumulated indicating that with practice observers can learn to reduce the interference caused by a salient distractor (Chelazzi et al., 2019; Geng et al., 2019; Liesefeld & Müller, 2019). Hence, Müller and colleagues revealed that the amount of interference caused by an irrelevant color singleton decreases as its overall rate of occurrence increases (Müller et al., 2009; Sauter et al., 2019), and similarly a singleton appearing in a frequent color is less distracting than one appearing in an infrequent color (Stilwell et al., 2019).

Rejection of a color-singleton distractor can also take place according to spatial coordinates, such that an irrelevant color-singleton interferes less where it occurs more frequently, also known as the *distractor-location effect* (e.g., Ferrante et al., 2018; Sauter et al., 2018; Wang & Theeuwes, 2018b; Zhang et al., 2019). This pattern of results, achieved via statistical learning of the distractor spatial statistics, is typically interpreted as evidence of suppression exerted at the most frequent distractor location to attenuate the singleton capacity to attract attention (for a review, see Luck et al., 2021), and depending on the specific conditions, spatial suppression would be exerted either at the saliency map level (e.g., Ferrante et al., 2018; Wang & Theeuwes, 2018b) or at some lower levels, such as in dimension-specific or feature-specific maps (e.g., Zhang et al., 2019).

However, the fact that attentional capture is attenuated as a distractor repeatedly appears at a given location does not, in itself, demonstrate that this effect is due to suppression. Rather, it simply shows that distractor interference is modulated by the spatial distribution of the distractor, which for some reason has lost its capacity to attract attention, and while spatial suppression is certainly a plausible explanation, alternative mechanisms could account for the same pattern. For instance, distractors could attract attention in proportion to how unexpected or surprising their occurrence is at a given location. This aligns with the idea that attention is captured by surprising events (Itti & Baldi, 2009), namely, that capture is regulated according to a “prediction error” principle, where the extent of capture reflects the discrepancy between the actual and expected probability of an event (also see Sokolov et al., 2002). According to this view, the grabbing power of a distractor would be modulated by expectation, without necessarily invoking suppression. Therefore, support for the suppression hypothesis must come from independent evidence, beyond the spatial modulation of distractor interference, which merely reveals a correlation between capture and distractor spatial probability.

The role of distractor suppression in capture modulation is further supported by findings showing that, on distractor-absent trials, processing of task-relevant information is particularly impaired at the most frequent distractor location, also known as the *target-location effect* (e.g., Ferrante et al., 2018; Goschy et al., 2014; Sauter et al., 2018, 2019; Valsecchi & Turatto, 2021; Wang & Theeuwes, 2018b; Zhang et al., 2019). The target-location effect would attest to the fact that across trials a suppression has been exerted where the distractor appears more frequently, thus tonically downmodulating the saliency map activity at the corresponding location, so that when the target happens to occur at the same location, its selection is slowed down compared to other locations. The effect of suppression at the distractor location can also be relatively long lasting, as it can be detected even when the distractor is no longer presented for hundreds of trials (Turatto & Valsecchi, 2021). This suggests enduring plasticity in the saliency map, which can influence processing of relevant information occurring at the previous distractor location, where activity remains suppressed compared to other locations.

Although impaired processing of relevant information at the color-singleton distractor location provides strong complementary indication of suppression, some previous studies have shown conditions in which the target-location effect does not emerge (Allenmark et al., 2019; van Moorselaar et al., 2021; Zhang et al., 2019). For example, Zhang et al. (2019) found that when the color of the singleton remained fixed rather than varying randomly across trials, the distractor-location effect was not accompanied by a target-location effect. The authors suggested that, under these conditions, participants learned to suppress the irrelevant singleton at a level below the priority map (i.e., in color-specific maps), thereby leaving target signals in the priority map unaffected. Another factor that affects the possibility to obtain evidence that distractor suppression impairs target processing seems to be the ratio between frequent and infrequent distractor locations. Lin et al. (2021) systematically manipulated this ratio and found that, although the distractor-location effect consistently increased with higher ratios, the target-location effect emerged only when the distractor was at least eight times more likely to appear at the frequent compared to the infrequent location. As the authors acknowledge, the theoretical reasons why the target-location effect disappears below a certain ratio remain unclear, especially under the assumption that suppression is responsible for the observed modulation of capture, as indicated by the distractor-location effect. As we argue here, we suspect that what may drive the target-location effect, namely the possibility of observing evidence of target-processing impairment at the distractor location, is not a question of the ratio between the probability of the distractor at frequent and infrequent locations, but rather the absolute distractor rate at a given location.

**Fig. 1:**
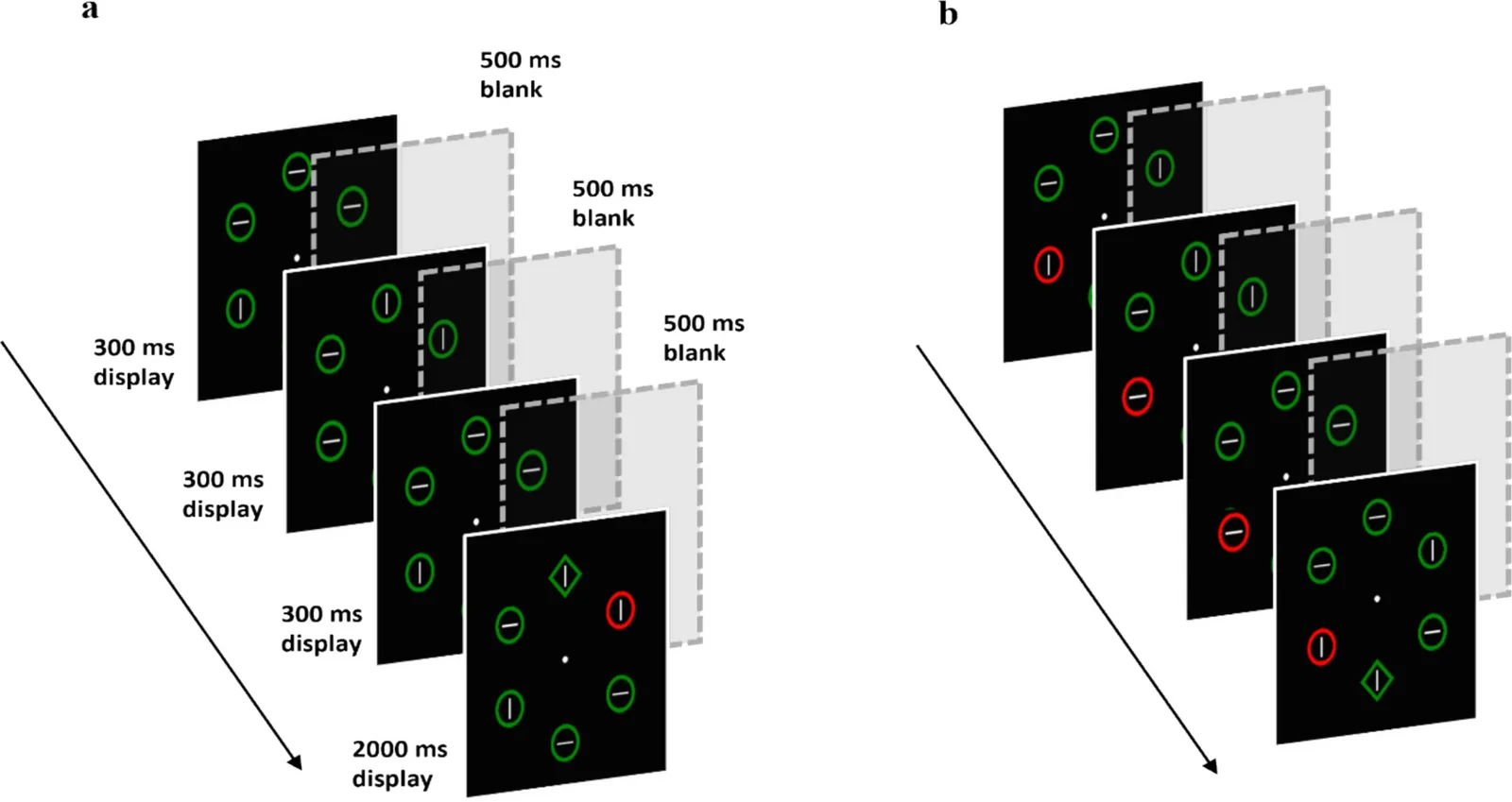
Schematic representation of the display sequence for the two distractor-present conditions. (**a**) Single distractor condition (40% of the trials): In the first three displays all the stimuli maintained the same shape (circles or diamonds) and color (green or red). In the last display both the target (odd-shaped figure) and the color-singleton distractor were present (the color singleton always in the same position across trials). (**b**) Repeated distractor condition (10% of total trials): In all four displays one of the stimuli was a color singleton appearing always in the same position within-trial (across the four displays) and across trials. The target appeared in the last display. The distractor-absent condition was the same as (a) but with no color singleton in the last display

In a recent study (Betteto et al., 2025) concerned with addressing distractor rejection in the absence of a target, we introduced a paradigm which was a modification of the standard additional singleton (Theeuwes, 1992), where each trial consisted of four consecutive discrete visual-search displays presented in rapid succession (see Fig. 1). The target singleton appeared only in the last display, whereas on distractor-present trials the salient color-singleton either appeared in all four displays (repeated condition) or just in the last one (single condition). In repeated and single trials, the distractor was presented at two distinct locations, which were fixed throughout the experiment. We decided to present four displays in each trial based on previous work by Wacongne et al. (2011), who studied the effects of prediction and expectation in the auditory domain. They used sequences of five sounds, in which they varied or omitted the last sound. In our case, we arbitrarily reduced them to four to keep the time of each trial more manageable while keeping a large enough number of presentations to form a strong expectation in trials when the distractor was repeated. Future experiments will be needed to address the role of the number of presentations. The main finding in our study (Betteto et al., 2025) was the observation of a strong capture attenuation in the repeated condition compared to the single condition, an effect which was also present in control experiments where in the repeated condition the position and color of the distractor were not kept constant. Based on our findings, we have tentatively hypothesized that distractor rejection could be achieved by means of different mechanisms (also see Valsecchi & Turatto, 2025), one driven by the rapid encountering of the distractor within a short time window, and another based on the across-trial distractor presentation and relying on spatial suppression. One observation that supported the idea of separate mechanisms was the lack of target impairment at the locations where the distractor previously appeared, although combined with a strong distractor rejection, a result that cannot be accounted for by the fact that the distractor color remained fixed across the within-trial repetitions. In particular, we propose that when the singleton was rapidly encountered within the same trial, namely within a limited time span, rejection was controlled by an expectation-based or prediction-error-based mechanism, as the one conceived in early models of habituation of the Orienting Reflex (OR) (Sokolov, 1963; Sokolov et al., 2002), and similarly by recent models of predictive coding. In Sokolov’s view, the attentional response to an irrelevant stimulus arises from a mismatch between the actual input stimulus and the expected one derived from the previous history of stimulation, stored in what Sokolov defined as the “neural model.” The larger the mismatch the stronger the OR, which also includes the covert attentional capture, and conversely the more a stimulus is predicted (or expected) the less it captures attention. This original idea was further elaborated in modern theories of predictive processing, where perception and attention are guided by backpropagation of prediction error (Feldman & Friston, 2010; Parr & Friston, 2017), and in particular with information theoretical models of salience, which postulate that attention is attracted by surprising stimuli (Baldi & Itti, 2010; Itti & Baldi, 2009). In line with this view, recent works have shown evidence of habituation of capture for both luminance transients and feature-singletons distractors (De Tommaso & Turatto, 2019, 2023; Turatto, 2023; Turatto & Valsecchi, 2023; Won & Geng, 2020).

The present work builds upon the pattern observed in Betteto et al. (2025): a robust attenuation of capture despite the absence of target-processing impairment. While this dissociation fell outside the scope of our previous study, it serves as the foundation for the current investigation. Hence, to further address the mechanisms underlying distractor rejection, as revealed by both the distractor-location and the target-location effect, here we carried out a set of dedicated experiments based on the same paradigm as in our previous study (Betteto et al., 2025).

## Experiment 1

The aim of the first Experiment was to address the efficacy of distractor rejection when the distractor is presented only in the last display compared to when the distractor also appeared in the preceding displays (within the same trial), in a more controlled and challenging fashion as compared to our previous study where the distractor rates in the two positions were the same (Betteto et al., 2025), resulting in a higher number of singleton presentations in the repeated condition compared to the single condition. Instead, in the present study the distractor rate at the repeated location was four times smaller than the distractor rate in the single location. The purpose of this manipulation was to expose participants to the same number of color singletons at the two locations. Note that with respect to the total number of trials, the rate (i.e., the probability of the distractor in a block of trials) of the single condition was 40%, whereas that of the repeated condition was 10%. Since on each distractor-present trial in the repeated condition the singleton appeared four times, one in each display, whereas there the singleton appeared only in the last display of the single condition, the total number of irrelevant color singletons presented in the two locations was the same.

Under these conditions, and based on previous findings with the standard additional singleton paradigm (Wang & Theeuwes, 2018b; Zhang et al., 2019), one should expect the strongest capture attenuation, with the relative evidence of suppression, at the single high-rate location compared to the repeated low-rate one, where capture, given the low distractor rate (10%), should be particularly robust. On the other hand, if our previous observations were to be confirmed, we might expect a different pattern of results, namely a weaker capture at the less frequent repeated location than at the most frequent single location. In addition, no evidence of target-processing impairment should emerge at the repeated location, compared to the locations never occupied by the distractor, despite the expected strong capture attenuation.

### Method

#### Participants

Participants were recruited online via the Prolific platform (Prolific Academic Ltd, Oxford, UK). Participants had to be between 18 and 40 years old, to be fluent English speakers, to have declared having normal or corrected-to-normal vision, and to be able to run the experiment on a desktop computer, which was mandatory. An a priori power analysis was conducted using G*Power 3.1.9.7 (Faul et al., 2007) to determine the minimum sample size required to test the study hypothesis. We expected less capture in the repeated condition compared to the single condition, based on what we observed in a preliminary set of experiments (Betteto et al., 2025). The effect was substantiated by a large effect size, but since there are not any other studies that employed the same paradigm as ours, here we chose to be conservative and based the analysis on a medium effect size. The results indicated that the required sample size to achieve 80% power for detecting a medium effect (*d* = 0.5), at a significance criterion of α =.05, was *N* = 27 (one-tailed *t*-test), which we rounded to 30. Participants were excluded for failing to reach the threshold of 80% of correct responses and were replaced by new participants (men; women; mean age). They all gave informed consent and were paid 7€/h for their participation. We did not collect information on the ethnicity of the participants. The experiment was carried out in accordance with the Declaration of Helsinki, and with the approval of the local institutional ethics committee (Comitato Etico per la Sperimentazione con l’Essere Umano, Università degli Studi di Trento, Italy).

#### Apparatus, stimuli, and procedure

The experiment was programmed in PsychoPy3 version 2022.2.5 software (Peirce et al., 2019) and hosted on the Pavlovia platform (Open Science Tools Limited, Nottingham, UK). Since participants may use different screen sizes, stimuli were defined in specific “height” units, allowing consistent automatic rescaling. Measures are reported in degrees of visual angle considering a monitor height of 34 cm and a viewing distance of 60 cm.

In each trial there were four consecutive displays, each presenting the following events (see Fig. 1): a white fixation dot (0.4° diameter) appeared against a black background in the center of the screen for 500 ms; then six geometrical figures appeared around the fixation point. They could be circles (2.5 ° diameter, 0.2° thick) or outlined diamonds (2.1° width × 2.1° height, 0.2° thick; due to a difference in how certain polygons are constructed by the software, width/height needed to be slightly different from diameter for the stimuli to appear of the same size). Each figure embedded either a vertical or a horizontal white line (1.5° length, 0.1° thickness). The orientation of the line inside each shape randomly changed across each display of a trial. The first three displays we presented for 300 ms, and they consisted of the same shape and color figures (either red or green), except for the color singleton when present. After each display there was a 500-ms blank interval. In the fourth and last display, which lasted 2,000 ms or until response, one of the figures was the shape singleton target (see Figs. 1a and 1b). The positions in which the distractor appeared were fixed across trials (counterbalanced across participants) and were located on the two opposing diagonals of the array of figures. The target always appeared in a random position throughout the experiment. Both shapes (circles and diamonds) and colors (red and green) randomly swapped across trials. Participants performed two blocks of 150 trials each, and the distractor was present on 50% of trials. Of these, 40% were single trials where the distractor appeared only in the last display (60 trials) and 10% were repeated trials where the distractor was presented in each display (15 trials). Thus, the distractor rate was 40% on the single trials and 10% on the repeated trials. Trials were presented always in a randomized order. Participants reported as quickly and accurately as possible the orientation of the line inside the target by pressing the right-arrow key on the keyboard if the line was horizontal, and the down-arrow key if the line was vertical. If a wrong response was given, or the time for responding was exceeded, the feedback “Wrong!” or “Try to be faster” appeared on the screen for 500 ms. A 500-ms blank inter-trial interval concluded the trial.

### Results and discussion

Analyses were performed in RStudio (Posit Team, 2024). RT analysis was performed on correct trials (mean overall accuracy = 90%). Recently, it has been highlighted how, for RT research, several types of outlier removal procedures (OEPs) could cause more problems than they might solve (Miller, 2023). Thus, here we chose to only remove RTs below 200 ms (<.001%), as they would be physically implausible for a task of this type.

#### Attentional capture

**Fig. 2:**
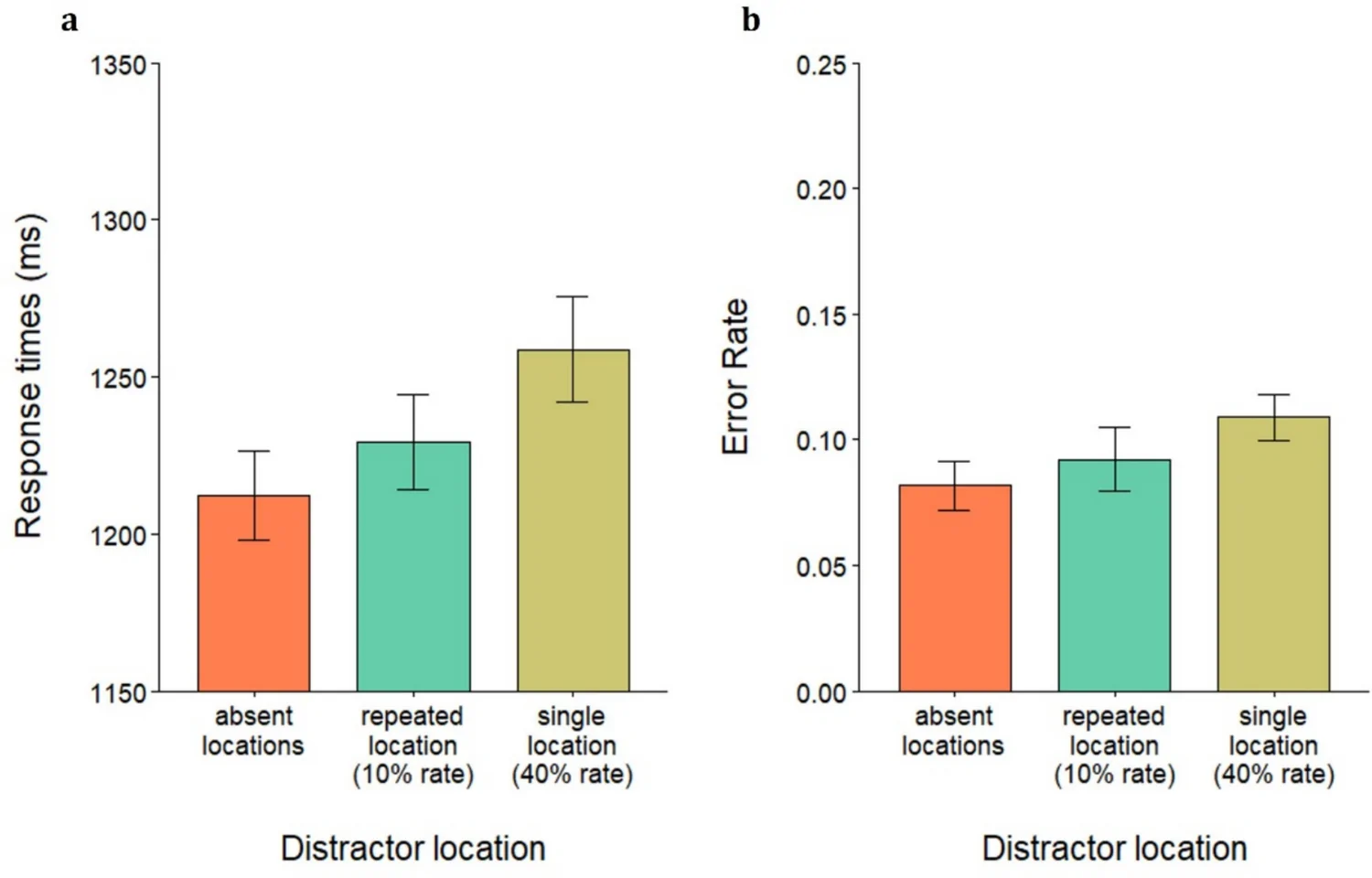
Attentional capture at the distractor locations. Mean response times (**a**) and error rate proportions (**b**) for each distractor location. Error bars reflect standard errors of the mean

An ANOVA was performed on mean correct RTs with the factor distractor condition (absent, repeated, or single), which was significant, *F*(2, 58) = 23.35, *p* <.001, ${\eta}_{p}^{2}$ =.4. Post hoc comparisons (which were subject to Holm correction here and in the following experiments) showed that at the single high-rate location (*M* = 1,258 ms; *SD* = 91), participants were significantly slower than both at the repeated low-rate location (*M* = 1,229; *SD* = 83), *t*(29) = 3.8, *p* =.001,* d* = 0.7, and in the distractor-absent condition (*M* = 1,212; *SD* = 78), *t*(29) = 7, *p* <.001,* d* = 1.3. The RT difference between the repeated low-rate location and the distractor-absent condition was significant, *t*(29) = 2.7, *p* =.01, *d* = 0.5 (see Fig. 2a). An ANOVA on the error rates revealed a main effect of distractor condition, *F*(1.52, 43.95) = 3.84, *p* =.04, ${\eta}_{p}^{2}$ =.01. Post hoc tests showed that the error rate was higher at the single high-rate location (*M* = 0.1; *SD* = 0.04) than in the distractor-absent condition (*M* = 0.08; *SD* = 0.05), *t*(29) = 3.55, *p* =.001, *d* = 0.6. No significant difference was found between error rates at the single high-rate and repeated low-rate locations (*M* = 0.09; *SD* = 0.06), *t*(29) = −1.36, *p* =.3, and between the distractor-absent condition and the repeated low-rate location, *t*(29) = 1.15, *p* =.3.

Capture was therefore largely reduced at the repeated low-rate location compared to the single high-rate location, despite in the latter the across-trial distractor rate being four times larger, while the number of color singletons appearing in the two locations was the same.

#### Target-processing impairment

**Fig. 3:**
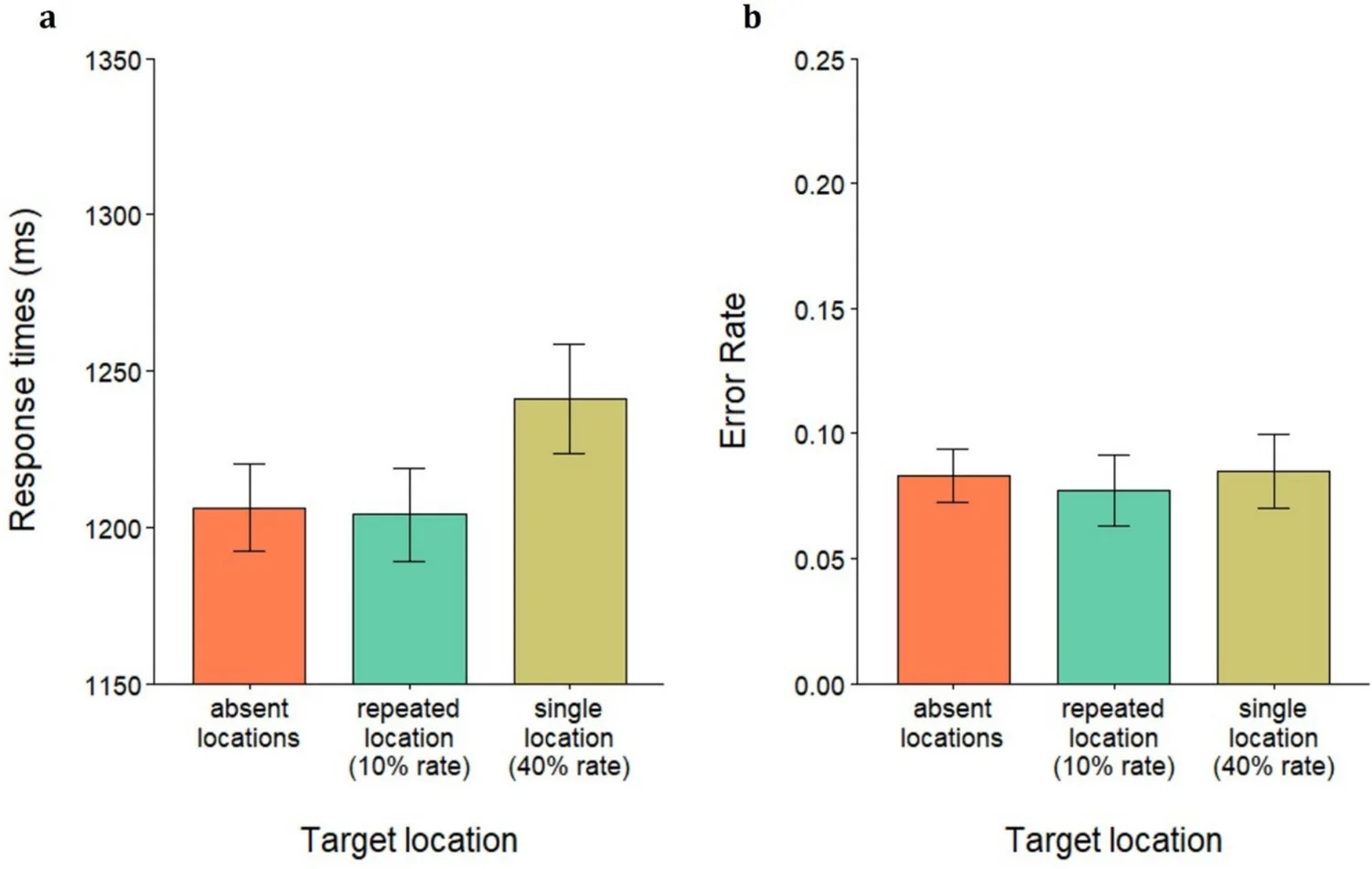
Target processing at the distractor locations. Mean response times (**a**) and error rate proportions (**b**) when the target appeared, in distractor-absent trials, in each of the two distractor locations and in the distractor-absent locations. Error bars reflect standard errors of the mean

We then analyzed the efficiency of target selection as a function of whether the target appeared, in distractor-absent trials, in either the repeated low-rate or the single high-rate location. An ANOVA on mean correct RTs revealed a main effect of target location, *F*(1.41, 40.88) = 10.57, *p* <.001, ${\eta}_{p}^{2}$ =.22. Post hoc tests showed that participants were slower when the target appeared at the single high-rate location (*M* = 1241; *SD* = 95) compared to both the repeated low-rate (*M* = 1,204; *SD* = 81), *t*(29) = 3.19, *p* =.007, *d* = 0.5, and the distractor-absent locations (*M* = 1,206; *SD* = 76), *t*(29) = 4.48, *p* <.001, *d* = 0.8. By contrast, RTs at the repeated low-rate location were comparable to those in the distractor-absent locations, *t*(29) = 0.2, *p* =.7, *d* = −0.05 (see Fig. 3a). An ANOVA on error rates showed no significant main effect, *F*(2, 58) = 0.16, *p* = 0.8.

To summarize, attentional capture was weakest where the color-singleton distractor was less likely to occur across trials, as the distractor appeared at the repeated location with a 10% rate whereas it occurred at the single location with a 40% rate. In addition, note that the different amount of capture found at the single and repeated locations was observed despite the total number of color-singleton presentations being the same in the two locations. This result, besides replicating our preliminary observations (Betteto et al., 2025), but in more controlled conditions, appears to be in sharp contrast with previous findings obtained with the classic additional-singledom paradigm, showing that capture is invariably weaker where the distractor appears at the highest rate (e.g., Wang & Theeuwes, 2018a, 2018b; Zhang et al., 2019). Finding the opposite result may suggest that the distractor-rejection mechanism based on within-trial repetitions is different from, and ultimately more efficient than, the mechanism based on across-trial distractor repetitions, as traditionally studied using the standard additional-singleton paradigm. Further indications that with the multiple-display paradigm we may have engaged distinct mechanisms for distractor rejection comes from the observation that target processing was more efficient where attentional capture was more strongly attenuated, namely at the repeated low-rate location compared to the single high-rate location. This is also in contradiction with previous findings attesting an opposite pattern, namely a stronger target-processing impairment where capture is weaker (e.g., Ferrante et al., 2018; Lin et al., 2021; Sauter et al., 2018; Wang & Theeuwes, 2018b, 2018a).

A plausible explanation for the lack of target-processing impairment at the repeated location could be the fact that at this location the distractor rate was too low (also see Lin et al., 2021). However, this alone does not yet explain why capture was much more strongly attenuated where the distractor was less likely to occur but repeatedly presented within the same trial. In the next experiments we further explored the possibility that this pattern of results might indicate the engagement of distinct rejection mechanisms operating on within-trial and across-trial distractor repetitions.

## Experiment 2

In Experiment 1, the target-processing impairment in distractor-absent trials (the putative sign of suppression) was found only at the single high-probability position, and not at the repeated low-probability position. What is the reason for this difference? As argued above, a possible explanation might be that the low across-trial distractor probability (10%) may have been insufficient to trigger the suppressive mechanism. This would indicate that suppression is linked to the across-trials probability of occurrence of the distractors, which needs to be high enough in order for the system to exert it. If this were the case, finding its signature (the target-processing impairment at the suppressed location) should be unrelated to the presence of within-trial repetitions. Alternatively, within-trial repetitions did in fact matter in that they either avoided suppression entirely and reduced capture via another mechanism, or if some type of suppression was exerted within-trial, it was too weak to leave a detectable trace in the saliency map. Either way, the reason for the lack of target-processing impairment at the repeated position would be found when the distractor was presented repeatedly within the same trial, irrespective of the across-trial probability.

To distinguish between these alternatives, in Experiment 2 everything was the same as Experiment 1, except that the distractor was repeated in the four displays in both locations, which, however, still differed in terms of the overall distractor rate across trials (10% vs. 40%). By introducing within-trial distractor repetitions at both locations, we should observe one of two scenarios. One possibility is that the target-processing impairment is once again found only in the high-probability location, despite the fact that distractors at that location are also repeated within-trial. This would show that target-processing impairment, and by extension, suppression, is related to across-trials distractor probability. On the other hand, if within-trial repetitions are the reason for the lack of target-processing impairment, then it should not be found in either distractor position, irrespective of across-trial probability.

### Method

#### Participants

Given that this experiment should highlight the role of the across-trial rate by equating the within-trial repetitions, we considered as the crucial comparison the difference in target-processing impairment between high- and low-probability locations, which in Experiment 1 was substantiated by a medium effect size (*d* = 0.5). We thus calculated our sample size as for Experiment 1. Thirty participants (17 men; 13 women; mean age 28.2 years) were recruited through Prolific, as in the previous experiment. Five did not reach the 80% accuracy threshold and were substituted with new participants.

#### Apparatus, stimuli, and procedure

These were as for Experiment 1, except that on distractor-present trials the distractor was presented repeatedly at both the high (40%) and the low (10%) probability locations.

### Results and discussion

#### Attentional capture

**Fig. 4:**
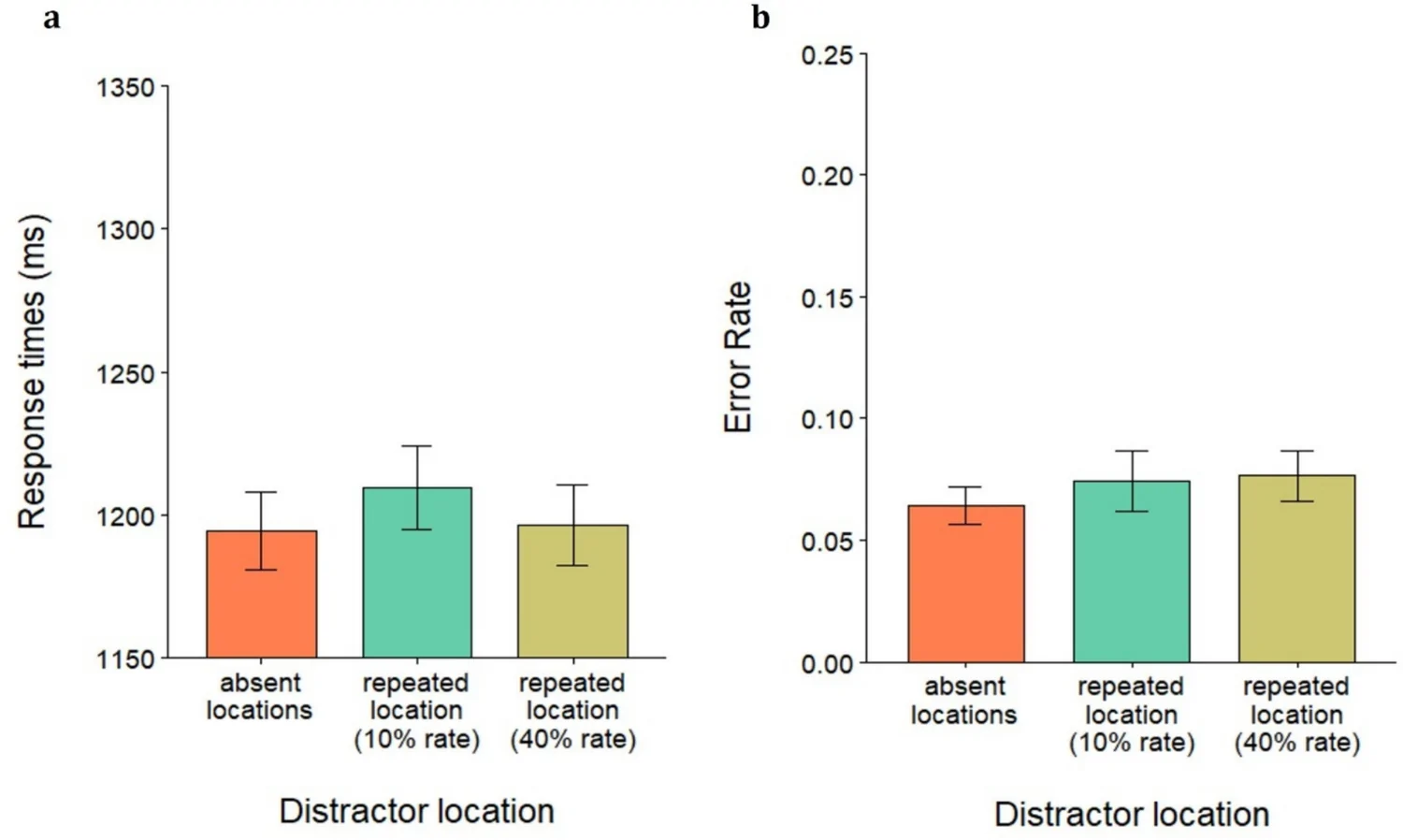
Attentional capture at the distractor locations. Mean response times (**a**) and error rate proportions (**b**) for each distractor location. Error bars reflect standard errors of the mean

All RT analyses were performed on correct trials (92% of total trials). After removal of RTs below 200 ms (<.001%), an ANOVA was performed on mean correct RTs with the factor Distractor condition (absent, high probability, or low probability), which was not significant, *F*(1.29, 37.29) = 3.31, *p* =.06, ${\eta}_{p}^{2}$=.1 (see Fig. 4a), suggesting that capture was effectively reduced both at the high-probability and at the low-probability locations. An ANOVA on the error rates found no effect of distractor condition, *F*(2, 58) = 1.35, *p* =.2, ${\eta}_{p}^{2}$=.04 (see Fig. 4b).

#### Target-processing impairment

**Fig. 5:**
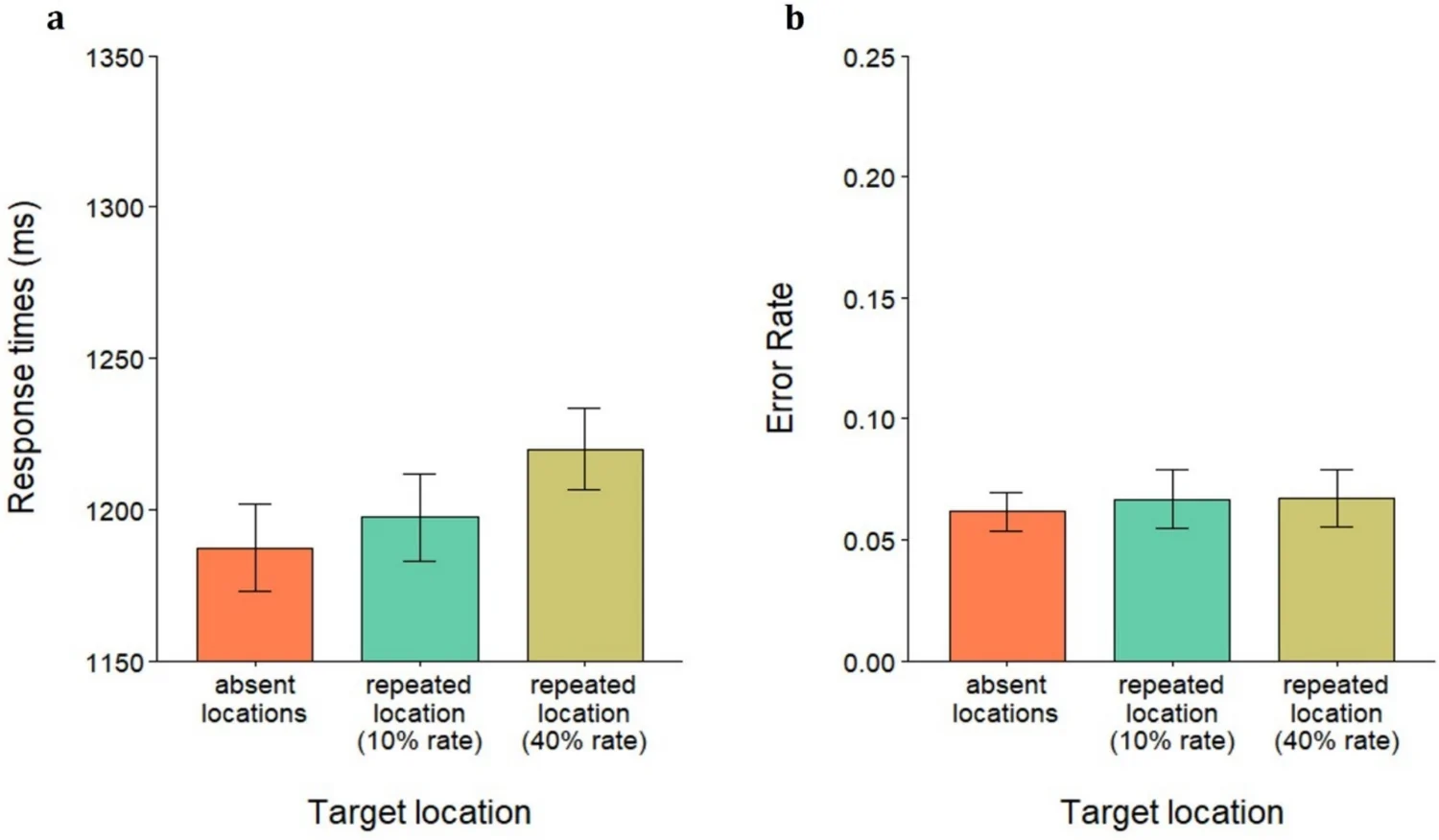
Target processing at the distractor locations. Mean response times (**a**) and error rate proportions (**b**) when the target appeared, in distractor-absent trials, in each of the two distractor locations and in the distractor absent locations. Error bars reflect standard errors of the mean

We compared RTs, in distractor-absent trials, when the target appeared at the high-probability distractor location, at the low-probability distractor location, or at the remaining locations where the distractor never appeared. An ANOVA on mean correct RTs revealed a main effect of target location, *F*(2, 58) = 6.84, *p* =.002 ${\eta}_{p}^{2}$ =.2. Post hoc tests showed that when the target was at the high-probability distractor location RTs were significantly higher than when it appeared at the distractor-absent locations (*M* = 1,187; *SD* = 79), *t*(29) = 3.95, *p* =.001, *d* = 0.7. No significant difference in RTs was found when the target appeared at the high-probability location (*M* = 1,219; *SD* = 73) compared to the low-probability location (*M* = 1,197; *SD* = 78), *t*(29) = 2.15, *p* =.07, *d* = 0.4. RTs at the low-probability location were comparable to those in the distractor-absent locations, *t*(29) = 1.23, *p* =.2, *d* = 0.2 (see Fig. 5a). An ANOVA on error rates showed no significant main effect, *F*(2, 58) =.15, *p* =.8.

With regard to our initial question, in the present experiment capture abolishment was again accompanied by a target-processing impairment only at the at the high-probability distractor location, whereas such impairment was lacking at the low-probability location. Since the distractor was repeated within-trials in both locations, the results indicate that the target-location effect strongly depends on the distractor occurrence across-trials, possibly engaging a robust spatial suppression at the distractor location when such probability is high enough, in line with previous findings obtained with the classic version of the additional-singleton paradigm (Allenmark et al., 2022; Wang & Theeuwes, 2018b; Zhang et al., 2019).

Importantly, these results revealed that when the distractor was repeatedly presented within trial at both at the high-probability (*M* = 1,196; *SD* = 76) and at the low probability (*M* = 1,209; *SD* = 80) locations capture was virtually abolished at both locations, as in both cases RTs did not differ from those observed in distractor-absent trials (*M* = 1,194; *SD* = 74). So, as compared to Experiment 1, where the lowest capture emerged at the repeated low-probability distractor location, a complete reduction of capture was also observed at the most likely distractor location provided that the distractor iteratively appeared, within the same trial, during the four displays. In Experiment 1, suppression built across trials was not sufficient to eliminate capture completely at the single high-probability position, although it left a detectable trace (the target-processing impairment). Capture was instead abolished at the high-probability position only when the distractor was repeated within the same trial, as documented by the present experiment. This indicates that the within-trial distractor repetitions engage a very efficient mechanism to attenuate attentional capture, which adds to the across-trial suppression but can work independently to a great effect, as attested by the reduction of capture in the low-probability position in both experiments.

## Experiment 3

Taken together, the previous experiments indicate that two independent but additive mechanisms contribute to distractor rejection. One takes effect across-trial, probably based on suppression and it yields target-processing impairment when probability of occurrence is sufficiently high; the other one reduces capture via within-trial distractor repetitions and can be extremely efficient even at low rates of occurrence. To further strengthen this conclusion, in the present experiment, as in Experiment 1, the distractor was repeated in one location (repeated condition) and was presented only in the last display in the opposite location (single condition). However, unlike Experiment 1, now the distractor rate was raised to 35% in both locations. Under these conditions, we expect to find evidence of target-processing impairment at both the repeated and the single locations. Crucially, however, based on the results of Experiments 1 and 2, we expect capture to be more strongly attenuated in the repeated condition as compared to the single one, despite the two sharing the same distractor rate across trials.

### Method

#### Participants

In the previous two experiments, the difference in target-processing impairment between the high-probability and the distractor-absent locations (the crucial comparison in this case) was substantiated by effect sizes of *d* = 0.5 (Experiment 1) and *d* = 0.7 (Experiment 2). We chose to be conservative and determined our sample size based on a medium effect size, with the same calculation as for previous experiments. Thirty participants (19 men; 11 women; mean age 33.2 years) were recruited through Prolific, as in the previous experiments. Seven did not reach the 80% accuracy threshold and were substituted with new participants.

#### Apparatus, stimuli, and procedure

These were all the same as in Experiment 1, except that the local distractor rate was 35% at both locations (the distractor was present in 70% of the total trials).

### Results and discussion

#### Attentional capture

**Fig. 6:**
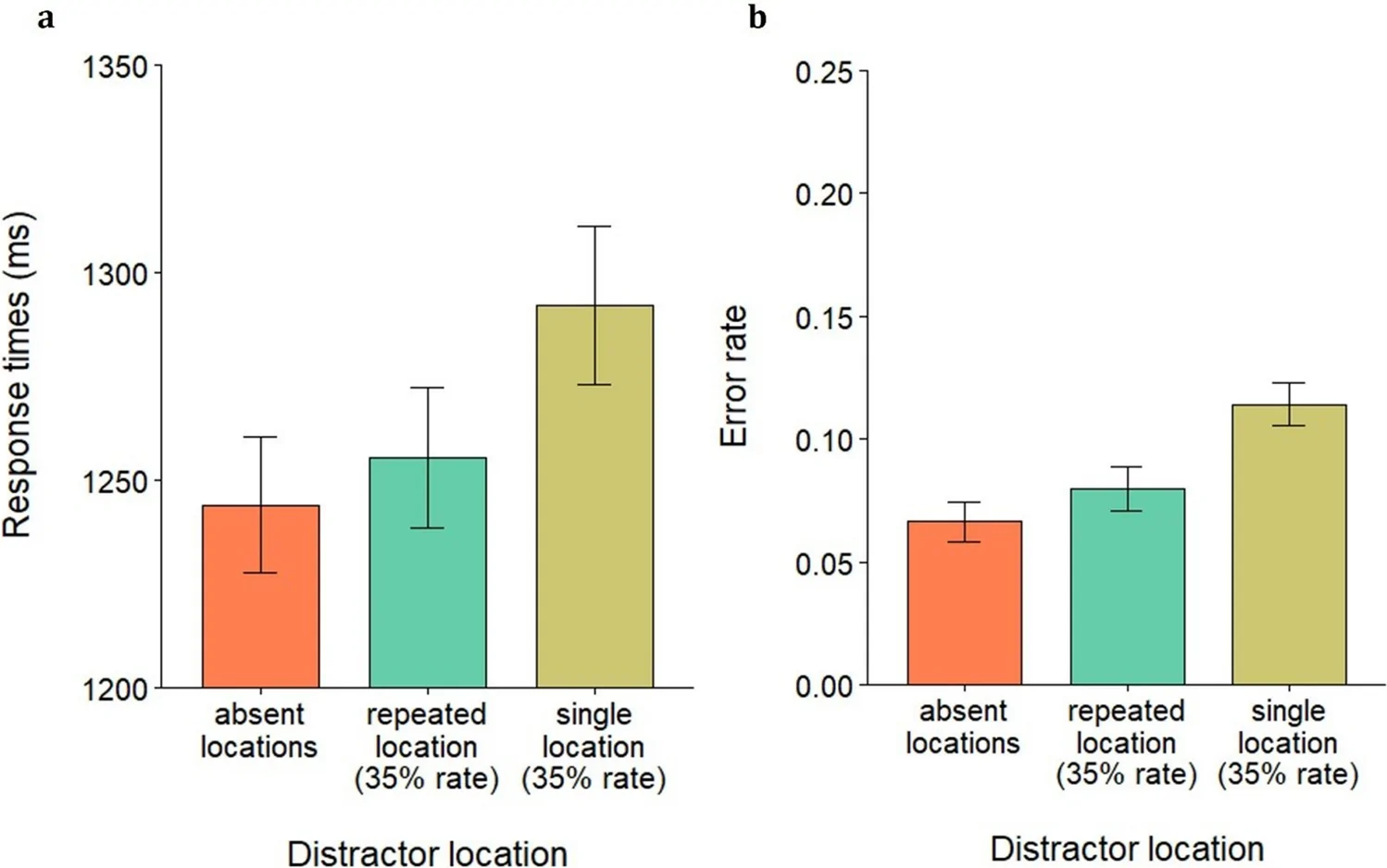
Attentional capture at the distractor locations. Mean response times (**a**) and error rate proportions (**b**) for each distractor location. Error bars reflect standard errors of the mean

All RT analyses were performed on correct trials (91% of total trials). After removing RTs below 200 ms (<.001%), an ANOVA was performed on mean correct RTs with the factor distractor condition (absent, repeated, or single), which was significant, *F*(2, 58) = 37.48, *p* = <.001 ${\eta}_{p}^{2}$ =.56. Post hoc comparisons showed that at the single location (*M* = 1,292; *SD* = 103) participants were significantly slower than at the repeated location (*M* = 1,255; *SD* = 92), *t*(29) = 5.71, *p* = <.001,* d* = 1, and in the distractor-absent condition (*M* = 1,244; *SD* = 89), *t*(29) = 7.62, *p* <.001,* d* = 1.4. At the repeated location they were also slower than in the distractor-absent condition, *t*(29) = 2.56, *p* =.01,* d* = 0.4, although by a smaller margin (see Fig. 6a). An ANOVA on the error rates found a significant main effect of distractor condition, *F*(2, 58) = 30.81, *p* = <.001, ${\eta}_{p}^{2}$=.5. Post hoc tests showed that error rates followed the same pattern of RTs with error rates in the single condition (*M* = 0.11; *SD* = 0.04) being the highest compared to the repeated condition (*M* = 0.07; *SD* = 0.04), *t*(29) = 4.76, *p* = <.001,* d* = 0.8, and the distractor-absent condition (*M* = 0.06; *SD* = 0.04), *t*(29) = 7.30, *p* = <.001,* d* = 1.3. Error rates in the repeated condition were higher than those in the distractor-absent condition *t*(29) = 2.79, *p* =.009,* d* = 0.5.

#### Target-processing impairment

**Fig. 7.**
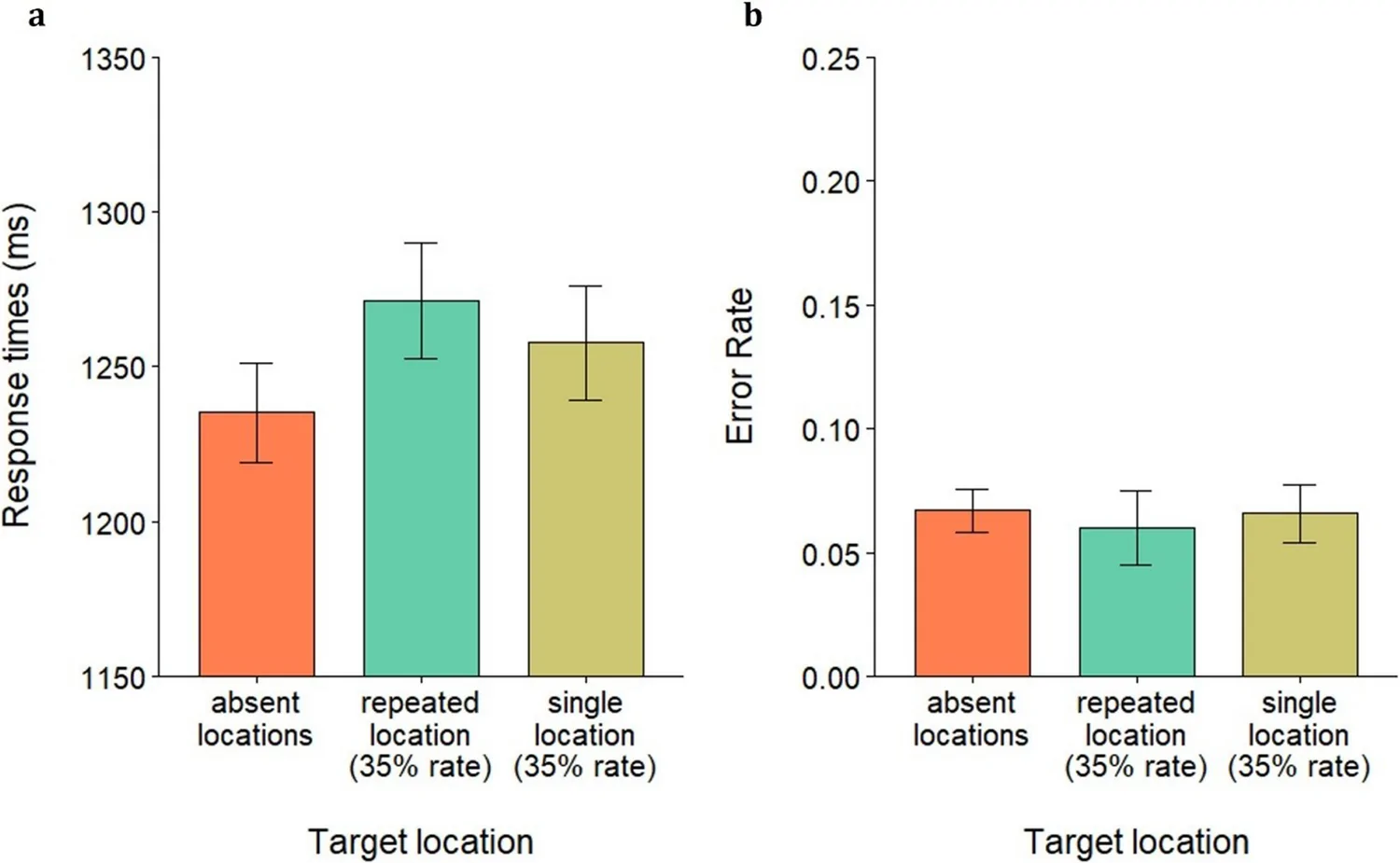
Target processing at the distractor locations. Note. Mean RTs (a) and error rate proportions (b) when the target appeared, in distractor-absent trials, in each of the two distractor locations and in the distractor absent locations. Error bars reflect standard errors of the mean.

We compared RTs, in distractor-absent trials, when the target appeared at the repeated distractor location, at the single distractor location, or in one of the remaining locations where the distractor never appeared. An ANOVA on mean correct RTs revealed a main effect of target location, *F*(1.44, 41.73) = 5.2, *p* =.01 ${\eta}_{p}^{2}$ =.1. Post hoc tests showed that participants were slower when the target appeared at the single location (*M* = 1,257; *SD* = 99) compared to the distractor-absent locations (*M* = 1,235; *SD* = 88), *t*(29) = 2.5, *p* =.03, *d* = 0.4. They were also slower when the target appeared at the repeated location (*M* = 1,271; *SD* = 93) compared to the other distractor-absent locations, *t*(29) = 3.7, *p* =.002, *d* = 0.7. RTs at the repeated location where comparable to those in the single location, *t*(29) = 0.9, *p* =.3, *d* = 0.1 (see Figure 7a). An ANOVA on error rates showed no significant main effect, *F*(1.48, 42.79) = 0.14, *p* =.8 ${\eta}_{p}^{2}$ =.005.

These results confirmed two important conclusions suggested by previous experiments: a) distractor rejection is accompanied by target-processing impairment at the corresponding location only when the distractor rate across trials is sufficiently high. If the target-location is taken as evidence that suppression is involved in distractor rejection, then such suppression is controlled by the across-trials distractor rate, regardless of whether or not the distractor is repeatedly presented within the same trial; b) on top of the across-trial rejection mechanism, repeating the distractor within the same trial provides a further advantage in controlling attentional capture, as attested by the fact that capture was clearly weaker at the repeated location, even when the distractor rate was lower than at the single location.

Overall, this pattern of results supports the hypothesis of two independent distractor rejection mechanisms: one operating on a longer time scale and based on distractor repetitions across trials, and possibly relying on spatial suppression, and a second one operating on a shorter time scale, based on rapid distractor repetitions. At present, it is unclear whether this latter mechanism also relies on suppression.

## Experiment 4

The aim of the present experiment was to gather direct evidence, within the same experiment, and from the same pool of participants, supporting the possible existence of distinct within- and across-trial distractor rejection mechanisms. To do so, we arranged three distractor-present conditions, each tied to a fixed location: repeated (5% local rate), single high-probability (40% local rate), single low-probability (5% local rate), plus the distractor-absent condition. Based on what we have observed in the first three experiments, we expected the following pattern of results concerning capture attenuation and evidence of target-processing impairment at the different locations.

First, we predicted capture to be most strongly attenuated (or even completely absent) in the repeated location, and this despite the very low local distractor probability of occurrence (5%), which appears insufficient to engage suppressive mechanisms. Second, capture should be the largest in the single low-probability location, where the distractor probability was also very low (5%) and therefore not engaging suppression; furthermore, the lack of within-trial repetitions precluded the corresponding rejection mechanism to be recruited. Third, we expected capture to be at some intermediate level in the single high-probability location because although the beneficial effects of the within-trial distractor repetitions are precluded, the distractor probability across trials should be sufficiently high (40%) to trigger the suppressive mechanism. Consequently, we predicted to find evidence of target-processing impairment only in this condition, and not in the other two where the across-trials probability would be too low (5%) for suppression to be engaged.

### Method

#### Participants

Regarding the effect size of target-processing impairment in the high-probability versus the other locations, the comparisons in the previous three experiments yielded effect sizes ranging from *d* = 0.5 to *d* = 0.8. Here we added one distractor condition increasing the complexity of the experiment. For this reason, we once again chose to be conservative in the selection of the effect size on which to base our sample size determination and chose to stay on the lower end of that range (i.e., medium effect size, *d* = 0.5). As in the previous experiments, this yielded *N* = 27, which we rounded to 30. Participants (16 men; 14 women; mean age 28.5 years) were recruited through Prolific, as in the previous experiments. Two did not reach the 80% accuracy threshold and were substituted with new participants.

#### Apparatus, stimuli, and procedure

**Fig. 8:**
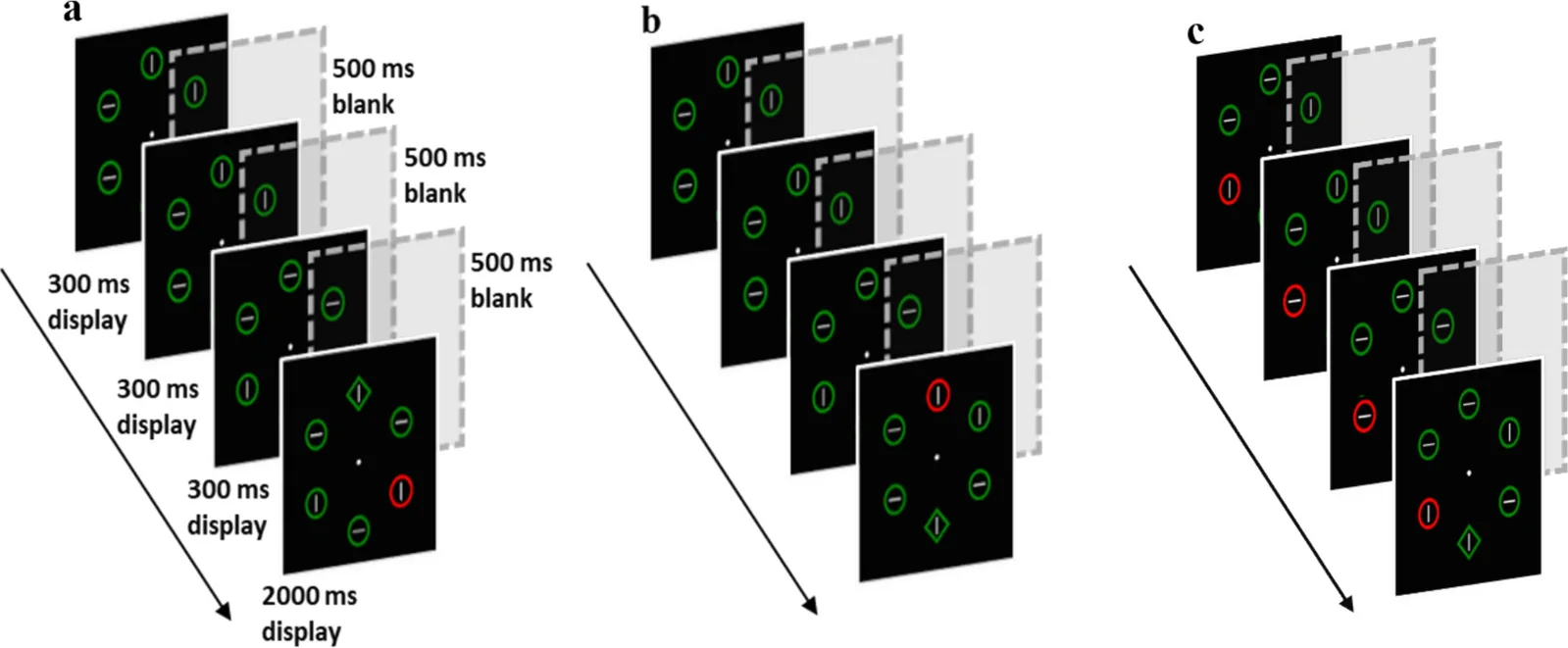
Display examples for the distractor-present conditions*.* (**a**) Single high-probability distractor condition (40% of the trials): In the first three displays all the stimuli maintained the same shape (circles or diamonds) and color (green or red). In the last display both the target (odd-shaped figure) and the color-singleton distractor were present (the color singleton was always in the same position across trials). (**b**) Single low-probability condition, which was the same as the single high-probability condition, only less frequent (5% of total trials). (**c**) Repeated distractor condition (5% of total trials): In all four displays one of the stimuli was a color singleton always appearing in the same position within trial (across the four displays) and across trials. The target appeared in the last display. The distractor-absent condition was the same as (a) and (b) but with no color singleton present in the last display

These were all the same as in the previous experiments, except for the following changes. There were three distractor-present conditions instead of two. Given the presence of two low-probability conditions, which consisted of very few trials, we increased the total number of trials to 450, split into three blocks of 150 each. The distractor-present trials were 50% of the total trials (225 out of 450) and were divided into the single high-probability (40%), the single low-probability (5%), and the repeated condition (5%). Each distractor condition was assigned to a position which remained fixed throughout the experiment and was equidistant from the other two in the array (120° apart). Positions were counterbalanced across participants Fig. 8.

### Results and discussion

#### Attentional capture

**Fig. 9:**
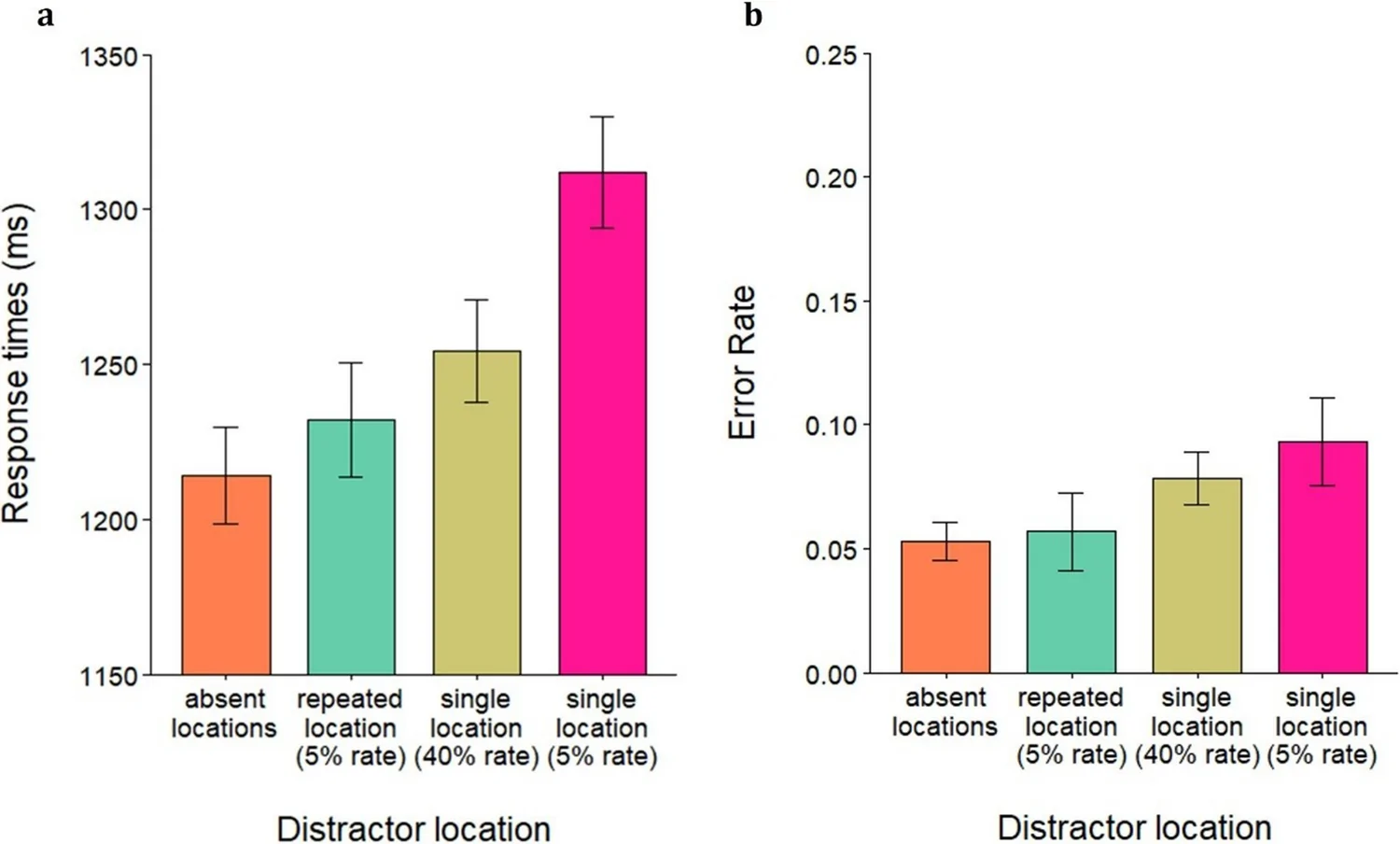
Attentional capture at the distractor locations. Mean response times (**a**) and error rate proportions (**b**) for each distractor location. Error bars reflect standard errors of the mean

All RT analyses were performed on correct trials (93% of total trials). No RTs were further removed from analyses, as they were all above 200 ms. An ANOVA was performed on mean correct RTs with the factor distractor condition (absent, repeated, single high-probability, single low-probability), which was significant, *F*(2.29, 66.29) = 51.53, *p* = <.001, ${\eta}_{p}^{2}$ =.6. Post hoc comparisons showed that participants were slower at the single low-probability location (*M* = 1,312; *SD* = 98) compared to the single high-probability location (*M* = 1,254; *SD* = 90), *t*(29) = 7.5, *p* <.001,* d* = 1.3, to the repeated location (*M* = 1,231; *SD* = 100), *t*(29) = 7.8, *p* <.001,* d* = 1.4, and to the distractor-absent condition (*M* = 1,214; *SD* = 86), *t*(29) = 11, *p* <.001,* d* = 2. At the single high-probability location participants were slower than in the distractor-absent condition, *t*(29) = 8.8, *p* <.001,* d* = 1.6. The difference between the repeated location and the distractor-absent condition was very close to being significant, *t*(29) = 2, *p* =.05,* d* = 0.3 (see Fig. 9a), as well as the difference between the repeated and single high-probability locations, *t*(29) = −1.98, *p* =.05, *d* = 0.4.

An ANOVA on the error rates showed a main effect of distractor condition, *F*(1.71, 49.5) = 4.8, *p* =.01, ${\eta}_{p}^{2}$=.14. Post hoc tests showed that the error rate at the single high-probability location (*M* = 0.08; *SD* = 0.05) was higher than those in the distractor-absent condition (*M* = 0.05; *SD* = 0.04), *t*(29) = 5.74, *p* <.001,* d* = 1. The error rate at the single low-probability location (*M* = 0.09; *SD* = 0.09) was also higher than both in the distractor-absent condition *t*(29) = 2.74, *p* =.01,* d* = 0.5, and at the repeated location (*M* = 0.05; *SD* = 0.04), *t*(29) = 3.56, *p* =.006,* d* = 0.6.

#### Target-processing impairment

**Fig. 10:**
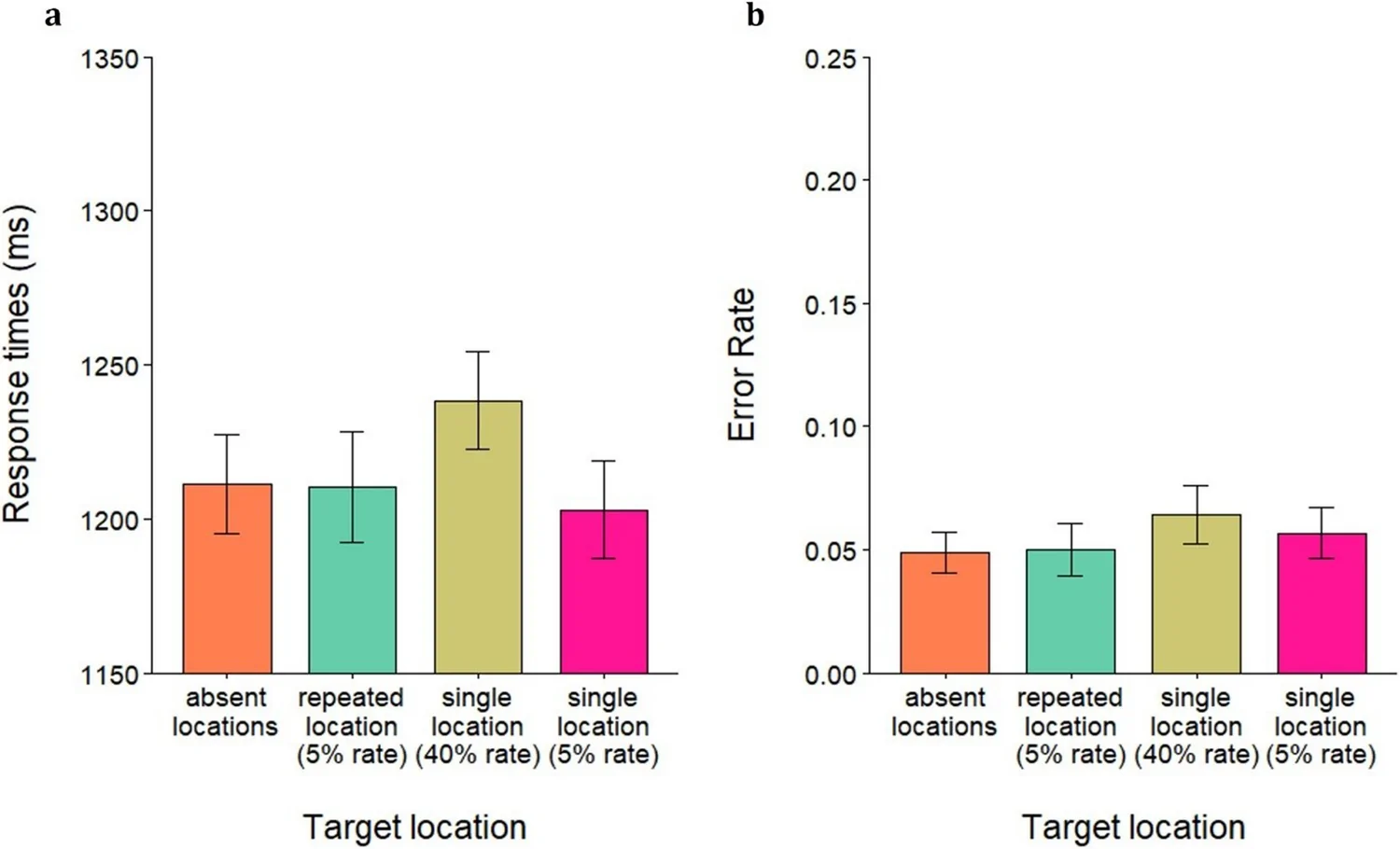
Target processing at the distractor locations. Mean response times (***a***) and error rate proportions (***b***) when the target appeared, in distractor-absent trials, at each of the three distractor locations and at the distractor-absent locations. Error bars reflect standard errors of the mean

We compared RTs, in distractor-absent trials, when the target appeared at the repeated location, at the single high- and low-probability location, or in the remaining locations where distractors never appeared. An ANOVA on correct RTs revealed a main effect of target location, *F*(3, 87) = 7.27, *p* <.001, ${\eta}_{p}^{2}$ =.2. Post hoc tests showed that participants were slower when the target appeared at the single high-probability location (*M* = 1,238; *SD* = 86) compared to the distractor-absent locations (*M* = 1,211; *SD* = 88), *t*(29) = 3.92, *p* =.002, *d* = 0.7, to the repeated location (*M* = 1,210; *SD* = 98), *t*(29) = 2.94, *p* =.02, *d* = 0.5, and to the single low-probability location (*M* = 1,203; *SD* = 87), *t*(29) = 4.5, *p* <.001, *d* = 0.8 (see Fig. 10a). The difference in RTs between the repeated location and single low-probability location was not significant, *t*(29) =.4, *p* =.9, *d* = 0.1. RTs at the distractor-absent locations and the single low-probability location were also not significantly different, *t*(29) = 1, *p* =.9, *d* = 0.1, as well as those at the distractor-absent locations and the repeated location, *t*(29) =.1, *p* =.9,* d* = 0.02. An ANOVA on error rates showed no significant main effect, *F*(2.37, 68) = 0.8, *p* =.4.

The results of Experiment 4 confirmed and consolidated the findings from the first three experiments. Both the reduction in capture and the pattern of target-processing impairment aligned with our predictions. The repeated condition produced the strongest attenuation of capture, despite its local low distractor rate (5% rate). Capture was also reduced at the single high-probability location (40% rate), though to a lesser extent, while the strongest capture occurred at the single low-probability location (5% rate). However, this linear pattern of capture was not mirrored in the pattern of target-processing impairment, which was absent in both the repeated and the single low-probability conditions, despite the stark difference in their levels of capture. In the single low-probability condition, the lack of target-processing impairment can be explained by the low distractor rate (5%), which agrees with the strong capture observed. Notably, the distractor rate was equally low in the repeated condition, yet capture was almost entirely abolished. This indicates that, given the same across-trial rate, the within-trial repetitions significantly enhanced distractor rejection and reduced attentional capture. Evidence of target suppression emerged only in the single high-probability condition, where the distractor rate was sufficiently high (40%).

#### Block analysis

**Fig. 11:**
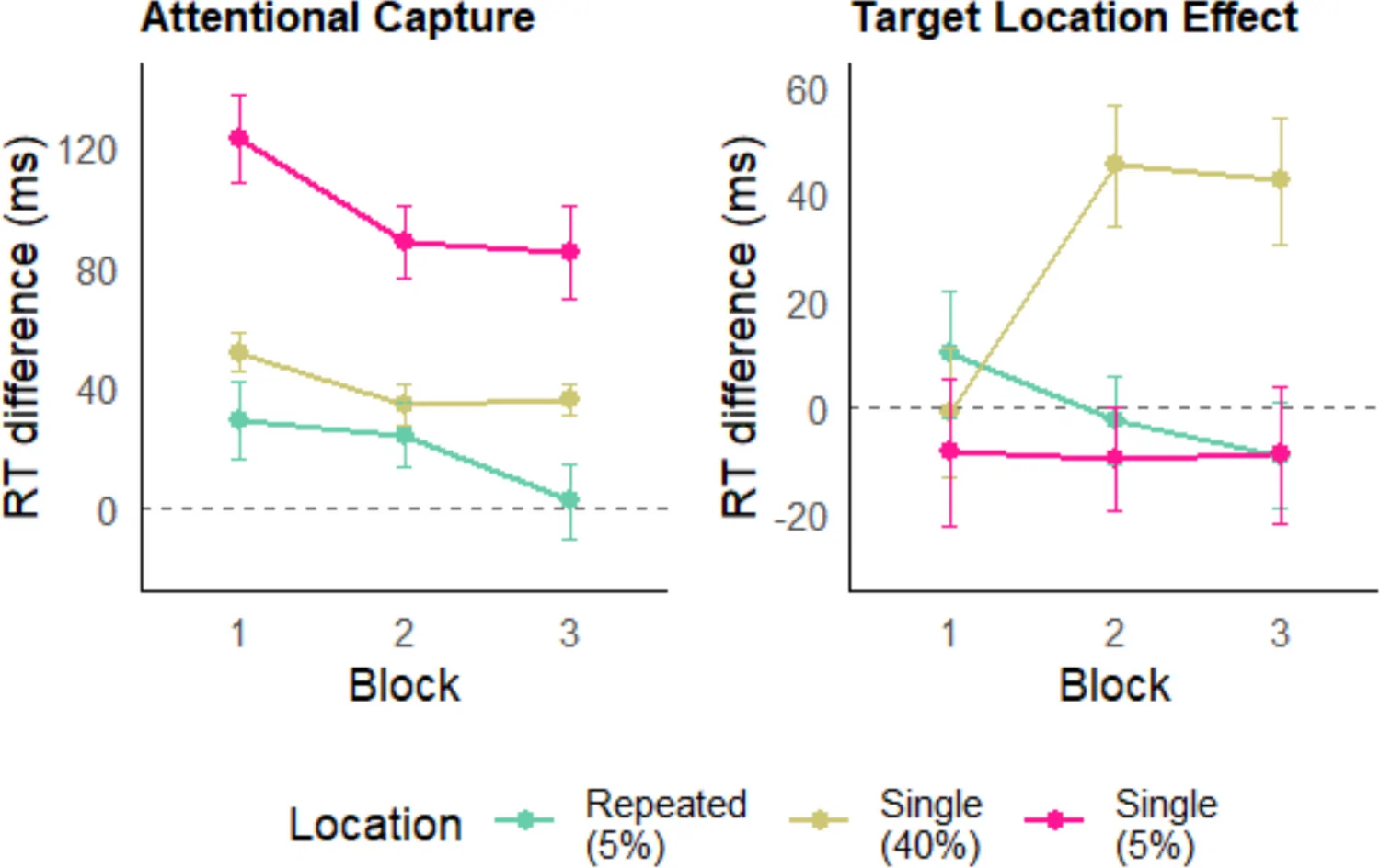
Attentional capture and target location effect at each distractor location across blocks. Capture is calculated by subtracting mean response times (RTs) in the distractor-absent condition from those of each distractor condition. Target Location Effect is calculated considering only the distractor-absent trials, by subtracting mean RTs when the target appeared in one of the non-distractor locations from the mean RTs when the target appeared in each of the distractor locations. Significance is reported from comparisons between the RT difference in block 1 and that in block 3, for each location, in the case of capture, and the RT difference when the target appeared at each distractor location in block 1 and that in block 3 in the case of target-location effect

To explore how the learning dynamics changed over time, in this experiment we also analyzed how attentional capture in each distractor condition (calculated by subtracting RTs in the absent condition from RTs in each distractor condition) changed across blocks. We then compared capture in Block 1 and Block 3 using paired *t*-tests. For all three conditions, capture was smaller in Block 3 (see Fig. 11, left panel); however, this difference reached significance only in the single high-probability condition (*t* = 2.14, *p* =.04), likely because the repeated and single low-probability conditions had a very small number of trials, making them statistically unreliable.

We also assessed how target impairment changed across blocks by subtracting RTs for targets in non-distractor locations from those in distractor locations across the three distractor-present conditions. In comparing impairment between Blocks 1 and 3, the single high-probability condition showed a significant increase (*t* = −2.54, *p* =.01), clearly visible from the second block onward (see Fig. 11, right panel), while no differences were found in the other two conditions. The pattern in the single high-probability condition aligns with the hypothesis of a slower across-trial distractor rejection mechanism whose detrimental effects on target processing accumulate over time, if there is a sufficient number of trials.

## General discussion

To further investigate distractor rejection mechanisms, in the present study we adopted a modified version of the additional-singleton paradigm, in which each trial consisted of four consecutive displays (Betteto et al., 2025). In the repeated condition the distractor appeared at the same location in each of the four displays but competed with the target only in the last one; in the single condition the distractor appeared, at a different location, together with the target only in the fourth display; in the distractor-absent condition no distractor was present in any of the four displays. In addition, the locations of the repeated and single conditions remained fixed during the experiment, leaving locations where the distractor never appeared to serve as a baseline condition to evaluate how target processing could be affected, if at all, by the distractor rejection mechanism involved.

In Experiment 1, where the color singleton appeared with a 10% rate at the repeated location, and with a 40% rate at the single location, the results showed the strongest capture attenuation at the repeated location, despite the distractor being four times less likely to appear here than at the single location. The same pattern of results was replicated in Experiment 4, where the distractor rate at the repeated location was even smaller (5%) compared to the single location (40%). This is clearly at odds with common findings, as previous studies with the standard additional-singleton paradigm have systematically reported that capture attenuation is proportional to the distractor rate at a given location, a phenomenon known as statistical learning of distractor locations (Allenmark et al., 2022; Chelazzi et al., 2019; Ferrante et al., 2018; Sauter et al., 2018; Wang & Theeuwes, 2018b; Zhang et al., 2019). It is therefore striking that in Experiments 1 and 4 the distractor with the lowest rate produced the least amount of interference, a result that replicated our initial observations with a similar paradigm (Betteto et al., 2025). Notably, here the results emerged despite participants, in Experiment 1, being exposed to the same total number of irrelevant color singletons at the repeated and single location, and even when the number of singletons was larger at the single location compared to the repeated one, as in Experiment 4.

The shared explanation for the observed capture attenuation in the additional-singleton paradigm is that suppressive activity is generated at the distractor location proportionally to its rate of occurrence (Goschy et al., 2014; Lin et al., 2021; Wang & Theeuwes, 2018b), which would attenuate the distractor saliency signals at the saliency map level (Ferrante et al., 2018; Gaspelin et al., 2015; Gaspelin & Luck, 2018; Itti & Koch, 2001; Krummenacher et al., 2009; Sauter et al., 2018; Zhang et al., 2019). In line with this view, it is also commonly found that in distractor-absent trials target processing is impaired (i.e., slowed down) at the high-probability distractor location, where the weakest capture is usually observed, which is interpreted as key evidence that this region has been suppressed (Ferrante et al., 2018; Lin et al., 2021; Sauter et al., 2018; Turatto & Valsecchi, 2021; Wang & Theeuwes, 2018a, 2018b; Zhang et al., 2019).

The results of Experiment 1 clearly showed, instead, that target processing was not impaired at the repeated distractor location, where capture was strongly diminished, whereas participants were slower when the target appeared at the single location. The same RT pattern was found in Experiment 4, where target-processing impairment was detected only for the single high-probability location. By contrast, because the distractor probability was very low (5% rate), no evidence of target suppression, and a strong capture, was found at the single low-probability location. However, it should be noted that the same lack of target suppression was also observed at the repeated location (same 5% rate), where the distractor was instead strongly rejected, as attested to by the fact that it failed to capture attention.

Hence, if the pattern of capture and target-processing impairment observed in the single condition is consistent with the engagement of a suppressive mechanism that attenuates the distractor’s ability to attract attention, as postulated by the suppression hypothesis (Gaspelin et al., 2025; Luck et al., 2021), the strong capture attenuation despite the lack of a target-processing impairment observed at the repeated location suggests that a distinct mechanism, different from the one operating across trials, may underlie distractor rejection when the distractor is repeatedly encountered within a short time window (see also Valsecchi & Turatto, 2025).

### Within-trial distractor repetitions: Possible rejection mechanisms

The first possibility worth considering is that at the repeated location participants may have used the within-trial repetitions to (rapidly) anticipate where the target would not appear, thereby excluding that location from search (the “excluded location” hypothesis). According to this view, participants would effectively search through one fewer location (five locations) than in the single condition (six locations), which could explain the reduced (or absent) capture in the repeated condition. There are several reasons for regarding this hypothesis as implausible. First, if it were true that in the repeated condition participants excluded one location from search, then RTs in this condition should be even faster than in the distractor-absent condition, where the search always involved six items. By contrast, RTs in the repeated and distractor-absent conditions did not differ. Second, since the target was a singleton, it is unlikely that search performance was meaningfully affected by whether the display consisted of six or (putatively) five elements. In fact, Gaspelin et al. (2015) reported faster RTs when the singleton was present rather than absent and used a similar argument to explain the singleton-presence advantage. However, in their paradigm (see Experiment 2), the target was not a singleton, thus requiring a serial search, one that could indeed benefit from excluding the singleton distractor from the search display. Another argument against the “excluded location” hypothesis comes from Experiment 2 of Betteto et al. (2025), where in the repeated condition, the distractor randomly changed position in each of the four displays within a trial, making it impossible to identify a location to exclude from search in the last display, in which the target also appeared. Nonetheless, a reduction of capture compared to the single distractor condition was found, demonstrating that repetitions were still effective despite not occurring in a fixed position. Considering all these reasons together, it seems therefore reasonable to dismiss the “excluded location” hypothesis.

Another possibility is that the strong capture attenuation found at the repeated location was still due to distractor suppression, but the lack of the target-location effect might simply result from the fact that the singleton appeared in the same color during the four within-trial display repetitions. Indeed, previous studies have reported no target impairment at the most frequent distractor location when the distractor color was blocked across trials (Zhang et al., 2019). In this case, the absence of a target-location effect has been explained by assuming that suppression was exerted at the level of dimension (or feature) maps, rather than at the saliency map level (Zhang et al., 2019). It is unlikely that in our Experiment 1 the distractor was suppressed but the target-location effect was absent because the distractor color was fixed, because in Experiment 2 evidence of target-processing impairment was found at the high-probability location, when the distractor color was fixed during the within-trial repetitions. A more concrete possibility is that evidence of target suppression can be found only when the across-trial rejection mechanism is involved, which, however, requires the distractor rate to be sufficiently high (also see Lin et al., 2021). In agreement with this possibility, in Experiment 3 a target-processing impairment at the repeated location was found when the corresponding distractor rate was increased to 35%. Regarding the evidence of suppression observed in the single high-probability condition (see Experiments 1 and 4), as attested to by the presence of the target-location effect, we must acknowledge that in our experiments the target was less likely to appear at this location compared to the other locations, among which is the repeated low-probability location. This imbalance in the probability of target appearance in certain positions could be taken into consideration when trying to explain the impaired target processing at the high-probability location. Namely, it could be that such slowdown in target processing may simply arise from a target-probability cueing, namely from the fact that the target appeared less frequently at the more frequent distractor location. However, Zhang et al. (2019) have already shown that even when accounting for target-probability cueing by rebalancing target-appearance probabilities, target-processing impairment is still evident at the high-probability distractor location. We therefore assumed that the target-processing impairment we observed in the present experiments could be interpreted, at least partially, as a sign of suppression. However, while suppression could still be responsible for the across-trial attenuation of capture, and for the related target-processing impairment at the high-probability location, the question remains open as to how the within-trial repetitions reduce attentional capture.

After having considered and ruled out the abovementioned explanations, we propose that when the singleton was rapidly encountered within the same trial, rejection was controlled by an expectation-based mechanism, as the one conceived in early models of habituation of the OR (Sokolov, 1963; Sokolov et al., 2002), and currently by recent models of predictive coding (Clark, 2013; Parr & Friston, 2019; Summerfield & de Lange, 2014) and information theoretical accounts of salience (Baldi & Itti, 2010; Itti & Baldi, 2009). Accordingly, in the present study the singleton at the repeated location was better ignored because it was largely expected (or unsurprising) given the within-trial repetitions, which would explain the significant reduction in capture despite the absence of target-processing impairment. Note that our proposal, that distractor rejection can be based on a “surprise” or prediction-error mechanism, could, in principle, also apply to distractor rejection in the classic additional-singleton paradigm, which consists of a single display and where suppression has typically been assumed to be the main mechanism (Luck et al., 2021). In line with the possibility that salient distractors may be ignored without necessarily invoking suppression (but see Gaspelin et al., 2025), recent studies have provided evidence suggesting that distractor rejection does not always require suppression (Kerzel et al., 2021, 2022; Kerzel & Huynh Cong, 2023; Oxner et al., 2023; Valsecchi & Turatto, 2024).

Although the “prediction error” mechanism we propose offers a parsimonious explanation at least for the strong capture attenuation found at the repeated location, we must acknowledge that at present we cannot completely exclude that suppression was still involved in this condition. However, unlike suppression based on distractor rate (e.g., Ferrante et al., 2018; Wang & Theeuwes, 2018b), whose effects are long lasting, detectable across trials, and to some extent even during extinction when the distractor is removed (e.g., Turatto & Valsecchi, 2023), such within-trial suppression, if any, would operate on a shorter timescale and thus be much more volatile. This putative form of “fleeting” suppression would rely on a very short-term memory system, leaving no trace beyond the specific trial event in which it is implemented. Still, even assuming that a fleeting suppression was operating at the repeated location, a crucial difference remains between the within-trial rejection mechanism and the across-trial one, because the former appears to be more efficient and independent of the distractor rate. Indeed, in Experiment 4 we found a complete capture attenuation at the repeated location and the strongest capture at the single low-probability location, despite the two conditions shared the same low distractor rate (5%).

### The possible role of priming, visual marking, and inhibition of return (IOR)

A further potential factor involved in capture attenuation when the distractor is repeatedly encountered, either across or within trial, is priming due to stimulus repetition. For example, Maljkovic and Nakayama (1994, 1996) originally showed that the selection of a salient target was more efficient when the target appeared in the same color or at the same location of the preceding trial. The beneficial effect of repetition also extends to distractors, and accordingly it has been found that capture is weaker when the distractor is repeated from one trial to the next, irrespective of any statistics (Goschy et al., 2014; Krummenacher et al., 2009; Qiu et al., 2023; Sauter et al., 2018). Goschy and colleagues (2014) ran three experiments to disentangle the contribution of the mere distractor repetition and of the distractor statistics (independently of any repetition), and found that both are involved in capture attenuation, yielding a larger effect when combined. Sauter et al. (2018) reported reduced interference when distractors repeated at the same location from trial to trial, and also found impairment of target processing when the target at trial N appeared at the distractor location at trial N-1. However, given the low distractor rate at the repeated location (5% or 10%), it is very unlikely that inter-trial priming of suppression may have played a role in capture attenuation in this case. So, inter-trial or across-trial priming cannot account for the strong capture attenuation observed at the repeated location, but, as discussed previously, if one assumes the presence of a fleeting suppression within trial (of which we do not have evidence), then priming may have occurred at the intra-trial level (i.e., during within-trial repetitions), thus strongly reducing capture in the repeated condition.

It is also worth noting that our paradigm bears some similarity to the paradigms used in the visual marking literature (e.g., Watson & Humphreys, 1997). In the repeated condition, the distractor is effectively previewed before appearing in the final display during target search. Prior work on visual marking has shown that previewing distractors can facilitate subsequent search when the previewed items reappear in the search display. Specifically, Watson and Humphreys (1997) asked observers to detect the presence or absence of a blue target under three conditions: (1) Single-feature: all distractors were blue. (2) Conjunction-feature: distractors were both blue and green. (3) Preview: a subset of green distractors was shown for 1,000 ms before the complete search display appeared. Search was more efficient in the Preview condition, comparable to the Single-feature condition, than in the Conjunction condition. Watson and Humphreys (1997) attributed this advantage to an inhibitory bias against previously presented items, which they termed visual marking. They proposed that the mechanism was top-down and goal-directed, relying on memory templates that coded for the locations (and possibly features) of the marked items. Subsequent work further supported the suppression account of visual marking. For example, Olivers et al. (2006) used an RSVP stream to induce an attentional blink before a preview-plus-search display. The visual marking effect emerged only when the preview display fell outside the blink window. Moreover, when a subsequent trial used previously previewed distractors as targets, search was impaired, suggesting that those items had indeed been suppressed and that suppression carried over across trials. More recently, ERP (event-related potential) studies have shown that previewed distractors elicit neural signatures typically associated with suppression (Berggren & Eimer, 2018).

Given these findings, one might argue that parallels exist between the visual marking paradigm and the multi-display additional singleton paradigm employed here. In both cases, preview displays can yield an advantage when the distractor is repeated in the final search display. However, despite this similarity, there are critical differences. First, although suppression has been demonstrated in visual marking, we did not find evidence of target-processing impairment, a hallmark of suppression, at the repeated location, despite strong capture reduction. Second, the sequence of events: in visual marking, the preview display is typically shown for 1,000 ms and is followed immediately by the search display, whereas in our experiments displays were separated by 500-ms blanks. This procedural distinction is important in light of Watson and Humphreys’ (1997) Experiment 6, where they presented distractors for 750 ms, inserted a 250-ms blank, and then showed the full search display. Under these conditions the preview advantage disappeared. By contrast, in the present study, as well as in Betteto et al. (2025), we observed an advantage from preview displays even with 500-ms blanks interleaved between displays. Taken together, these observations suggest that the preview advantage reported in visual marking studies and our results do not entirely overlap.

One might also wonder whether the reduced capture observed in the repeated condition could be an instance of IOR, which has been the focus of an extensive body of research spanning several decades (Klein, 2000). More recently, it has been noted that an increasing number of explanations, mechanisms, and theoretical accounts have been proposed with regards to IOR, making it the subject of diverse interpretations and highlighting the lack of a unified theoretical framework (Dukewich & Klein, 2015). This lack of consensus makes it difficult to draw firm conclusions about whether, and to what extent, the present findings are related to IOR, particularly in the absence of ad hoc experiments. Interestingly, however, in an attempt to provide a unified account of IOR, Dukewich (2009) argued that IOR may reflect habituation of spatial orienting, as originally conceptualized by Sokolov (1963). If so, and if IOR is indeed understood as a case of habituation governed by prediction-error attenuation mechanisms, then it would be appropriate to describe the capture attenuation observed in the repeated condition as an instance of IOR.

### Across-trial and within-trial rejection mechanisms

The finding that capture was more strongly attenuated in the repeated condition than in the single condition, despite the distractor being less frequent in the former, together with the observation that target-processing impairment emerged only at the single location, suggests that distractor rejection was probably governed by distinct mechanisms operating on different time scales in the two conditions.

The results of Experiments 2 and 3 are also informative with respect to the interplay between the within-trial and across-trial rejection mechanisms (also see Valsecchi & Turatto, 2025). As the findings of Experiment 2 indicate, when the distractor was repeated within the same trial at both locations, and consequently the within-trial rejection mechanism was engaged equally, capture attenuation was stronger when the distractor rate was higher (40%), namely when the across-trial suppression was involved. Accordingly, in distractor-absent trials evidence of target-processing impairment emerged at the high-probability (40%) location, but not at the low-probability (10%) location. Conversely, Experiment 3 showed that if the distractor rate was relatively high (35%) at both the repeated and the single location, the across-trial rejection mechanism operated at both locations, and target processing was slowed down equally as compared to the remaining locations.

Relevant for our hypothesis that distractor rejection mechanisms could operate on different time scales (also see Valsecchi & Turatto, 2025 for onset distractors) is a recent study by Qiu et al. (2024). The authors employed the classic additional-singleton paradigm and compared two conditions: a high-volatility condition where distractor-present trials were likely to appear in alternation with distractor-absent trials, and a low-volatility condition where distractor-present and distractor-absent trials appeared in streaks, without opposite-type trials interrupting their sequences. In both cases, there was distractor-probability cueing, with distractors appearing much more likely in one area of the display. The results showed that in both the high- and the low-volatility environments, probability cueing of distractor location had the usual effect, promoting strong rejection at the most likely location. Crucially however, when searching for target-processing impairment at the high-probability distractor location this was reliable only in the high-volatility condition, whereas it did not emerge in the low-volatility condition (Qiu et al., 2024). Note that in the low-volatility condition distractors were presented in a fashion similar to the repeated condition of the present study, in which we also did not find impairment of target processing when the distractor rate was low. To account for their findings, the authors hypothesized the existence of a mechanism that triggers suppression based on a dynamically adjusted threshold, set according to distractor probability and volatility. To achieve an efficient distractor rejection while reducing the number of “missed” rejections (i.e., lack of suppression in a distractor-present trial), a low threshold would be set in high-probability *and* -volatility distractor conditions, even at the cost of generating target-processing impairment; conversely to reduce false alarms (i.e., suppression in distractor-absent trials) a higher threshold would be set in the low-volatility conditions (Qiu et al., 2024). Broadly speaking, this notion of two types of rejection-handling applied to different conditions resonates, in principle, with the view we proposed here, which can be summarized as follows.

Recently Valsecchi and Turatto (2025) exposed participants to either one or multiple irrelevant visual transients (thus acting as distractors) at the same or different locations in the display before the occurrence of the target item. The results were interpreted as indicating that capture by onset distractors might be controlled by a within-trial habituation mechanism, operating on a shorter time scale and attenuating capture for repeated transients that recur at the same location within a brief temporal window; in addition to this, capture would be controlled also by a different mechanism dealing with the local probability of irrelevant transients across trials (see also Turatto & Valsecchi, 2023). In a similar fashion, overall the present results suggest the existence of two rejection mechanisms: one would function on the basis of the within-trial distractor repetitions, producing a very strong distractor rejection, without leaving traces of suppression, either because suppression is not involved, and capture is simply controlled by distractor expectation (Itti & Baldi, 2009; Sokolov et al., 2002; Turatto & Valsecchi, 2023), or because suppression is extremely volatile and remains confined within the same trial episode; the other one is the well-studied mechanism based on the distractor statistics across trials, which relies on suppression and generates target-processing impairment if the distractor rate is high enough. The two mechanisms can operate independently in reducing capture, but their effects can also be additive.

Crucially, the present study showed that the brain can engage both short-term (within-trial) and long-term (across-trial) memory-based rejection mechanisms, allowing them to operate concurrently at different locations to protect target selection from distractor interference Table 1.

**Table 1:** Summary of capture and target-location effect for each experiment

	Distractor location	Rate	Capture (ms)	Target impairment (ms)
Exp. 1	Repeated	10%	17 **	−2
	Single	40%	46 ***	35 ***
Exp. 2	Repeated LP	10%	2	10
	Repeated HP	40%	15	32 ***
Exp. 3	Repeated	35%	11 **	36 **
	Single	35%	48 ***	22 *
Exp. 4	Repeated	5%	17	−1
	Single LP	5%	98 ***	−8
	Single HP	40%	40 ***	27 **

Capture and target impairment (target-location effect) for each experiment and distractor location. Capture is calculated by subtracting mean response times (RTs) in the distractor-absent condition from those of each distractor condition. Target impairment is calculated considering only the distractor-absent trials, by subtracting mean RTs when the target appeared in one of the non-distractor locations from the mean RTs when the target appeared in each of the distractor locations. Significance is reported from comparisons between RTs at each distractor location and those at the distractor-absent condition, in the case of capture, and RTs when the target appeared at each distractor locations compared to the non-distractor locations in the case of target-location effect. *p <.05. **p <.01. ***p <.001

## Data Availability

Trial level data for all experiments has been made available and can be accessed online at https://osf.io/k7bfe/overview?view_only=1bedd941a6364ade81409fd0ea775f0c

## Declarations

### Open Practices Statement

None of the experiments were preregistered. Trial level data for all experiments has been made available and can be accessed online at https://osf.io/k7bfe/overview?view_only=1bedd941a6364ade81409fd0ea775f0c
